# Ocular Mucous Membrane Pemphigoid Demonstrates a Distinct Autoantibody Profile from Those of Other Autoimmune Blistering Diseases: A Preliminary Study

**DOI:** 10.3390/antib13040091

**Published:** 2024-11-14

**Authors:** Yingzi Liu, Lei Bao, Dharm Sodha, Jing Li, Adrian Mansini, Ali R. Djalilian, Xiaoguang Li, Hua Qian, Norito Ishii, Takashi Hashimoto, Kyle T. Amber

**Affiliations:** 1 Department of Developmental and Cell Biology, University of California Irvine, Irvine, CA 92617, USA; 2 Department of Dermatology, Rush University Medical Center, Chicago, IL 60612, USA; 3 Illinois Eye and Ear Infirmary, College of Medicine, University of Illinois at Chicago, Chicago, IL 60612, USA; 4 Central Laboratory, Dermatology Hospital of Jiangxi Province, Dermatology Institute of Jiangxi Province, and the Affiliated Dermatology Hospital of Nanchang University, Nanchang 331332, China; 5 Department of Dermatology, Kurume University School of Medicine, Kurume University Institute of Cutaneous Cell Biology, Kurume 830-0011, Japan; 6 Department of Dermatology, Graduate School of Medicine, Osaka Metropolitan University, Osaka 545-8585, Japan

**Keywords:** ocular pemphigoid, pemphigus, pemphigoid, autoimmune blistering disease, proteomics, antibody profiling, immunobullous

## Abstract

**Background**: Ocular predominant mucous membrane pemphigoid (oMMP) is a severe subtype of autoimmune blistering disease (AIBD), which can result in scarring and vision loss. The diagnosis of oMMP is challenging as patients often have undetectable levels of circulating autoantibodies by conventional assays. Likewise, the principal autoantigen in oMMP has been an area of debate. **Methods**: In this preliminary experiment, we performed Phage Immunoprecipitation Sequencing (PhIP-seq) on sera from patients with oMMP, as well as non-ocular MMP, bullous pemphigoid, and mucocutaneous-type pemphigus vulgaris. **Results**: We identified several autoantigens unique to oMMP relative to other AIBDs. We then cross-referenced these antigens against previously published single-nuclei datasets, as well as the International Mouse Phenotyping Consortium Database. Several protein hits identified in our study demonstrated enriched expression on the anterior surface epithelia, including TNKS1BP1, SEC16B, FNBP4, CASZ1, GOLGB1, DOT1L, PRDM 15, LARP4B, and RPL6. Likewise, a previous study of mouse knockout models of murine analogs CASZ1, HIP1, and ELOA2 reported that these mice showed abnormalities in terms of the ocular surface and development in the eyes. Notably, PhIP-seq failed to identify the canonical markers of AIBDs such as BP180, BP230, desmogleins 1 and 3, or integrin β4, indicating that the patient autoantibodies react with conformational epitopes rather than linear epitopes. **Conclusions**: oMMP patients demonstrate a unique autoantibody repertoire relative to the other AIBDs. Further validation of the identified autoantibodies will shed light on their potentially pathogenic role.

## 1. Introduction

Ocular predominant mucous membrane pemphigoid (oMMP) is a severe and rare subtype of autoimmune blistering disease (AIBD) [1,2,3]. oMMP is characterized by IgG and/or IgA autoantibodies against the structural proteins on the ocular basement membrane zone (BMZ), manifesting as chronic cicatrizing conjunctivitis [3]. Given the high risk of progression to permanent scarring and blindness, sometimes even in the absence of significant active inflammation, proper diagnosis is essential [4,5]. Although direct immunofluorescence (DIF) represents a gold standard in diagnosis, almost half of oMMP cases present with negative results. The diagnosis of MMP as a whole relies on the examination of DIF, indirect immunofluorescence (IIF), the enzyme-linked immunosorbent assay (ELISA), and/or immunoblot and histology. However, for oMMP specifically, histopathological and clinical exclusion of differential diagnoses is sufficient for diagnosis in the case of negative DIF and/or serologic workup. This distinction from the general MMP diagnostic guidelines reflects the high rate of false negative DIF regarding oMMP [3,6], as well as the clinical observations demonstrating comparable disease progression in cases with positive or negative DIF results [7,8,9,10].

The detection of circulating autoantibodies using IIF or ELISA can be helpful as a confirmatory test or for subtyping in the setting of a positive DIF. However, the sensitivity of these tests, particularly in patients with negative DIF, is very limited [11]. As oMMP is caused by the deposition of autoantibodies against the ocular BMZ, improved detection of these autoantibodies in circulation could significantly aid in diagnosis or disease monitoring in a non-invasive manner. 

The integrin β4 (ITGβ4) subunit was originally described as a specific autoantigen in oMMP [12,13,14]. While the original studies demonstrated the pathognomonic nature of the autoantigen, the validation studies have been less convincing. Only a segment of oMMP patients were noted to have ITGβ4 reactivity, and many patients with non-ocular AIBDs, such as in bullous pemphigoid (BP), demonstrated ITGβ4 reactivity. Thus, even if reactivity against ITGβ4 induces blistering, it does not appear to be ocular specific, as previously described [6,11,15,16,17,18,19,20,21,22,23,24]. 

In general, AIBDs are typically described as targeting a single antigen, with autoantibodies to secondary antigens acquired via epitope spreading, i.e., desmoglein 1 (Dsg1) in mucocutaneous-type pemphigus vulgaris (PV) or BP230 in BP [25,26]. The existence of BP with only BP230 antibodies [27] or mucosal PV with Dsg1 antibodies points towards a greater complexity [28]. Indeed, in PV, several non-desmoglein autoantibodies targeting other structural proteins or other organelles have been identified [29,30]. Autoantibodies to the calcium pump protein encoded by the ATP2C1 gene, C5a receptor, and HLA-DRA, as well as desmocollins 1(Dsc1) and Dsc3, have been demonstrated in a significant portion of PV patients [29]. While it is unlikely that all these additional autoantibodies are pathogenic or synergistic, some pathogenicity has been confirmed in several antigens. For example, the autoantibodies towards Dsc3 [31,32,33,34,35,36,37,38,39], M3 muscarinic acetylcholine receptor (M3AR) [40,41,42,43], and secretory pathway Ca^2+^/Mn^2+^-ATPase isoform 1 (SPCA1) [40] corresponded with disease activity in patients with PV, and their pathogenic roles were demonstrated both in vitro and in vivo [40]. Anti-mitochondrial antibodies were also demonstrated to be synergistic with anti-desmoglein antibodies [44]. Thus, the contribution of autoantibodies against these noncanonical antigens raises questions as to a monopathogenic or multipathogenic process [45]. Given the inconsistency in autoreactivity against ITGβ4 in oMMP, as well as its apparent lack of specificity for causing ocular disease, we hypothesized that multiple synergistic autoantibodies exist, leading to oMMP.

To test this hypothesis, we utilized Phage Immunoprecipitation Sequencing (PhIP-seq). PhIP-seq is a technology relying on a T7 peptidome phage display library to cover the entirety of the human proteome utilizing overlapping linear peptide fragments [46,47]. It has been used previously to demonstrate autoantibody profiles in patients with multiple sclerosis, type 1 diabetes mellitus, rheumatoid arthritis [48], autoimmune polyendocrine syndrome type 1 [49], membranous nephropathy [50], paraneoplastic neurological syndrome [51,52], COVID-19 [53,54], pediatric paraneoplastic Rapid-onset Obesity with Hypothalamic Dysfunction, Hypoventilation and Autonomic Dysregulation (ROHHAD) [55], inflammatory bowel disease [56], and interstitial lung disease [57]. In the present preliminary study, we utilized PhIP-seq to identify novel autoantibodies in a cohort of patients with oMMP and compared them with the results in other AIBDs.

## 2. Materials and Methods

### 2.1. Human Samples

Patients with diagnoses of non-ocular anti-BP180-type MMP, mucocutaneous-type PV (mcPV), BP, and oMMP were included. mcPV and BP sera reactive against both BP180 and BP230 were chosen, respectively, to increase the likelihood of epitope spreading against epithelial antigens. Diagnoses were based on current consensus diagnostic criteria, with sera collected at the initial time of diagnosis [3,58,59]. Sera from a subset of oMMP were tested by immunoblotting for ITGβ4 reactivity as previously described [16]. The results of serologic testing are summarized in Table 1. All patients provided written informed consent.

### 2.2. PhIP-Seq HuScan Library Construction

Briefly, we utilized the HuScan PhIP-seq library (CDI Laboratories, Mayagüez, PR, USA). *E.-coli*-codon-optimized oligonucleotides encoding 49-mer amino acid peptides with 25-mer overlaps that cover the entirety of the RefSeq human proteome were cloned en masse into a modified T7-Select vector (Millipore, Burlington, MA, USA). This resulted in fusion of the encoded 49-mer peptide to the T7 phage capsid with flanking FLAG and STREP tags. The library was then packaged, expanded, and assessed by Illumina sequencing.

### 2.3. PhIP-Seq

PhIP-seq was performed as previously described [45]. Briefly, 0.2 μL of serum was added to 0.5 mL PBS containing the HuScan library at an average of 105 excess clones per unique phage from the input library. Sera and phage libraries were incubated overnight. Protein A/G-coated magnetic beads were subsequently added and incubated at 4 h to pull down phage-bound IgG. Protein A/G beads in the absence of sera were used as a negative control (n = 36). Normal pooled plasma or anti-glial fibrillary acidic protein (GFAP)-antibody-spiked plasma (10 μg and 2 μg) was used as additional technical control. Protein A/G beads were subsequently washed and resuspended with Herculase II Fusion Polymerase master mix and amplified by PCR, whereby multiplex barcodes and sequencing adapters were incorporated. Barcoded samples were then pooled and sequenced using an Illumina sequencer. 

### 2.4. Bioinformatic Analysis

Analysis of the PhIP-seq data was performed as previously described [46]. Briefly, following demultiplexing, reads were aligned to the human peptide library, requiring a minimum aligned score of 50. Mapped reads to each phage clone were subsequently counted. EdgeR [59] was then used to estimate changes and *p*-values against the negative serum-free Protein A/G control. Log2fold increases of at least 2 and −log10(*p*-value) > 4 were used to define significantly enriched hits. False discoveries were controlled by assessing enriched hits in any of the negative control samples. For downstream analysis, clones corresponding with any epitope of the protein were pooled to demonstrate Boolean reactivity against the protein, regardless of epitope. Hits in the group of interest versus the rest of the disease groups were then compared using 2 × 2 matrices by Fisher’s exact test in R (4.1.0). The significance for Fisher’s exact test was set at *p* < 0.05. Heatmap of proportions of patients with significant hits of targeted antigens from each comparison cohort were generated in R based on transformed Z-scores. Uniform manifold approximation and projection (UMAP) visualization of clustering nuclei derived from corneoscleral wedge (CSW) tissue was generated using the Broad Institute’s Single Cell Portal [60]. Dot and feature plots showing the expression of significant hit genes in oMMP across all CSW cells were generated using the Broad Institute’s Single Cell Portal. Significantly targeted antigens in oMMP compared to other AIBD diseases underwent gene set enrichment for Gene Ontology using Enrichr [61]. Venn diagrams comparing enriched targeted antigens identified from our PhIP-seq study and the study by Kalantari-Dehaghi et al. [29] were generated in R. 

## 3. Results

### 3.1. PhIP-Seq Analysis of AIBD Sera Identified Distinct Targeted Antigens in oMMP Patients

**Figure 1:**
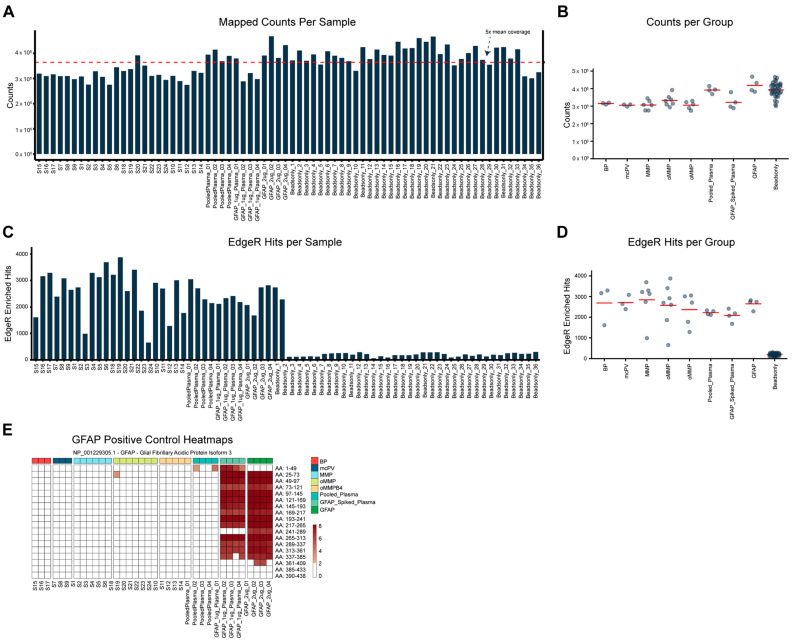
Quality control of PhIP-Seq data. (**A**) Number of mapped counts for each sample. The red dashed line indicates the level of 5× mean coverage. (**B**) Number of mapped counts for each group. (**C**) Number of peptide EdgeR hits called in each sample. (**D**) Number of peptide EdgeR hits called in each group. (**E**) Heatmap of pipeline-called anti-GFAP (glial fibrillary acidic protein) positive control hits in all samples.

The PhIP-seq analysis of the AIBD sera identified distinct targeted antigens in oMMP patients: more than 95% of all the samples, including the assay controls, achieved more than 1× coverage of the parent library and passed mapping quality control (Figure 1A). The mapped counts per sample and disease group as well as EdgeR hits per sample and disease group are shown in (Figure 1A–D). The enrichment of the GFAP antigen in the GFAP-positive control groups demonstrated the specificity of the targeted antigen detection by the assay (Figure 1E).

**Figure 2:**
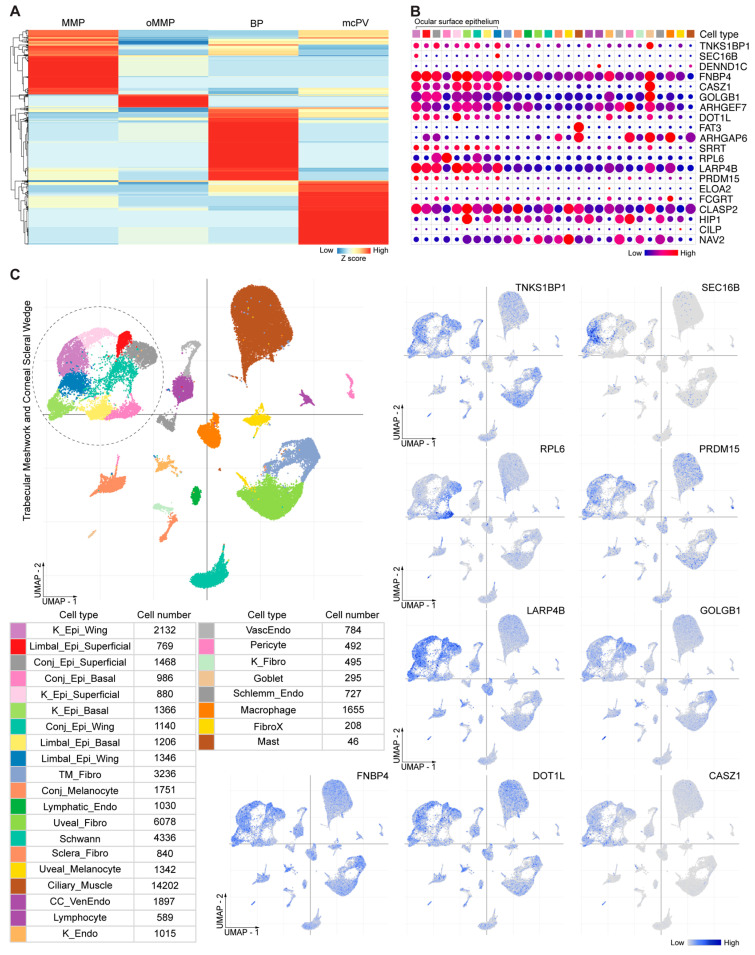
Distinct autoantibodies in various AIBD sera. (**A**) Heatmap of distinct autoantibodies defined from Fisher exact test on protein hits in the group of interest vs. controls. (**B**) Dot plot showing the expression of the top twenty distinct autoantibodies in MMP compared to other AIBD diseases in corneoscleral wedge (CSW) cells. Cell types were annotated at the top and color-coded the same as in panel (**C**). (**C**) UMAP visualization of clustering of nuclei derived from CSW tissue (top left). Cell type and cell numbers were shown (bottom left). Feature plots showing expression of selected top gene identified in MMP compared to other AIBD diseases in all CSW cells (right). Ocular surface epithelium subtypes are outlined.

The statistical analysis revealed that each disease group had unique targeted antigens compared to the other AIBD groups (Fisher’s exact test; *p* < 0.05). Specifically, 94 targeted antigens were significantly enriched in oMMP, 206 in BP, 182 in non-ocular MMP, and 186 in mcPV (Figure 2A; Supplementary Tables S1–S5). Notably, while the PhIP-seq assay demonstrated clustering based on disease states, reactivity against key extracellular autoantigens (type XVII collagen, Dsg1, Dsg3, and ITGβ4) in any of the disease groups was absent, except for BP230, an intracellular autoantigen. The top 20 hits in oMMP vs. the other AIBDs are shown in Table 2.

We next assessed the expression of the top 20 most significant targeted antigens in oMMP among all the human CSW cells from previously described human anterior segment single-nuclei sequencing datasets [60]. We investigated antigens enriched in either epithelial cells including the corneal (basal, wing, superficial, and transit-amplifying cells), limbal (basal, wing, and superficial), conjunctival (basal, wing, and superficial), or goblet cells, as well as fibroblasts. We identified enrichment of TNKS1BP1, SEC16B, FNBP4, CASZ1, COLGB1, DOT1L, PRDM 15, LARP4B, and RPL6, particularly in epithelial cells (Figure 2B,C).

We next cross-referenced the significantly targeted antigens with those from a prior study assessing the phenotypes in over 4000 gene knockout mouse models, identifying 347 genes causing ocular phenotype [62]. To our surprise, none of these overlapped with the genes of the targeted antigens that we identified. We then investigated additional ocular phenotypes in the International Mouse Phenotyping Consortium Database, in which *Casz1* was associated with the persistence of the hyaloid vascular system, Dot1l was associated with abnormal retina vasculature, and Hip1 was associated with abnormal cornea morphology and corneal opacity. Notably, of our top 20 targets, *Arhgap6*, *Fnbp4*, *Srrt*, *Rpl6*, *Prdm15*, *Fcgrt*, and *Clasp1* knockouts have not been phenotyped. ELOA2 does not have a murine analog; however, *Eloa* knockout results in abnormal eye morphology, anophthalmia, and abnormal vitreous body morphology [63]. 

### 3.2. Pathway Enrichment Analysis Demonstrated Enriched Signals in oMMP

**Figure 3:**
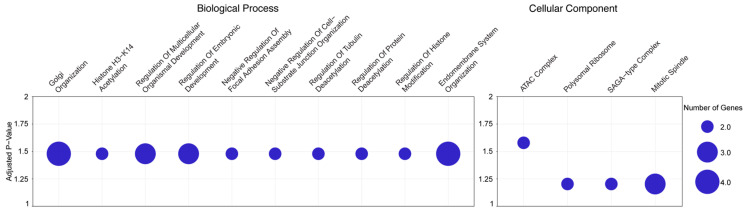
GO analysis of top autoantibodies enriched in oMMP patient sera. Bubble plots showing the enriched terms of biological process (**left**) and cellular components (**right**) in MMP compared to other AIBD diseases. Adjusted *p*-value is indicated in y-axis. Size of the bubbles indicates the number of genes enriched in each term.

To better understand the large-scale and potentially synergistic pathways targeted by upregulated antibodies in oMMP relative to the other AIBDs, we analyzed the Gene Ontology enrichment of gene symbols for targeted proteins enriched in oMMP (Supplementary Table S6). This analysis revealed the enrichment of twenty biological process terms, with the top ones related to the regulation of Golgi organization, histone acetylation, multicellular organismal development, embryonic development, focal adhesion assembly, cell-substrate junction organization, and tubulin deacetylation. Additionally, we identified four enriched cellular component terms, including the ATAC complex, polysomal ribosome, SAGA-type complex, and mitotic spindle (Figure 3; Supplementary Table S7).

### 3.3. Comparison to Prior Pemphigus Proteome Arrays

**Figure 4:**
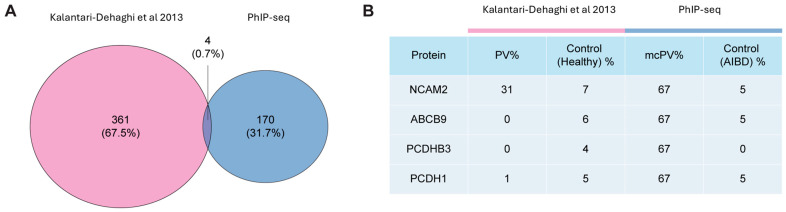
Autoantibodies enriched in mcPV patient sera. (**A**) Venn diagram representing the numbers and overlapping of autoantibodies identified from PhIP-seq in the current study and the study by Kalantari-Dehaghi et al. [29]; (**B**) Table of four overlapping autoantibodies between the two studies and proportion of patients showing autoantibodies in disease and control groups.

Given the prior large-scale screening of autoantibody reactivity in PV, we sought to utilize our preliminary dataset to perform a comparison of the PhIP-seq performance versus the traditional protein microarray [29]. The previous study was performed utilizing 701 human genes expressed in DH5α *E. coli* cells to identify autoantibodies in patients with PV relative to controls [28]. We thus compared the significant targeted antigens from this study with the ones defined in our mcPV patients relative to other blistering diseases. To our surprise, only four proteins demonstrated shared hits (NCAM2, ABCB9, PCDHB3, and PCDH1) between the two technologies (Figure 4; Supplementary Table S8). Notably, NCAM2, which was significantly targeted in the mcPV sera in both studies, was not expressed on the CSW. Further, Kalantari-Dehaghi and colleagues utilized a healthy control group, whereas we utilized only patients with various AIBDs with a much smaller sample size [29], which limits drawing major conclusions.

## 4. Discussion

In this preliminary study using well-characterized cohorts of patients with four AIBDs, including oMMP, non-ocular anti-BP180-type MMP, BP, and mcPV, we assessed the reactivity of sera by PhIP-seq and compared the results in oMMP with those of the other AIBDs. oMMP demonstrated a distinct autoantibody repertoire from those in non-ocular MMP, as well as those in BP and mcPV. Several hits demonstrated enriched expression in terms of the gene expression on the anterior surface epithelia, including TNKS1BP1, SEC16B, FNBP4, CASZ1, GOLGB1, DOT1L, PRDM 15, LARP4B, and RPL6. Likewise, a study on the mouse knockout models of murine analogs CASZ1, HIP1, and ELOA2 reported abnormalities regarding the ocular surface and development in the eyes of these knockout mice. Further direct validation of the pathogenicity of autoantibodies targeting these markers is, however, needed.

The targeting of intracellular antigens in other AIBDs has been previously observed, such as anti-BP230 antibodies in BP [27], and several non-desmoglein autoantibodies in PV [30,40]. In PV, the pathogenicity of the antibodies targeting the mitochondria [44,64], as well as the Golgi apparatus [40], has been demonstrated in vitro and in vivo. Thus, autoantibodies targeting cytoskeletal and organelle components, as we identified in oMMP, could exert significant pathologic effects. This could also account for the significant rate of negative DIF staining against the BMZ in patients with oMMP [3,6,11]. Thus, rather than an issue of sensitivity or proteolysis of deposited anti-BMZ antibodies, synergistic antibodies targeting intracellular antigens may alternatively induce separation. 

Our study has several notable limitations. Firstly, PhIP-seq appears to have a limited ability to identify classical extracellular antigens. Notably, patients with BP and mcPV each lacked reactivity with BP180 and Dsg1/Dsg3, respectively, using the PhIP-seq assay despite positive ELISA reactivities with these autoantigens. This is likely due to PhIP-seq’s inability to detect conformational epitopes as it utilizes short linear overlapping peptide fragments. This may also account for a discrepancy in the autoantibody profiles of patients with PV utilizing a traditional microarray approach versus PhIP-seq. Recent developments in proteomic technology, such as the development of Molecular Indexing of Proteins by Self-Assembly (MIPSA) [65], may improve high-throughput screening for autoantibodies as this technology utilizes in-vitro-expressed whole-recombinant proteins. However, detection limitations due to protein folding, transmembrane protein solubility, or post-translational modifications may still be present with this approach. 

Another limitation of this study is the small sample size and use of a diseased control. Our hypothesis was that a unique autoantibody profile would be present in oMMP. By using a control of patients with AIBDs, we sought to preliminarily assess the assay’s performance. Likewise, we hypothesized that autoantibodies unique to the oMMP relative to the other AIBDs would likely be more specific and relevant than those in healthy controls as the sera from patients with AIBDs likely demonstrates significant epitope spreading against epithelial antigens [26,66]. Thus, disease controls, particularly those demonstrating epitope spreading, were selected for increased specificity at the expense of the sensitivity of the epithelial antigens that may be missing in a healthy control population. Additionally, while ITGβ4 reactivity appears to be inconsistent and non-specific in oMMP overall [11], 10 of our 12 oMMP samples demonstrated reactivity with ITGβ4 by immunoblotting [15]. Thus, we were unable to perform a subgroup analysis to determine whether ITGβ4-negative patients share similar autoantibodies. 

## 5. Conclusions

In conclusion, in this preliminary study, we demonstrated a unique autoantibody profile in patients with oMMP relative to other AIBDs. While the assay had limited utility in identifying the canonical markers of AIBD, we identified numerous autoantigens uniquely targeted or significantly more targeted in oMMP than other forms of AIBD. Several of these antigens are expressed on the anterior ocular epithelium and have described pathologic implications from the results of knockout mouse studies. The validation of the pathogenicity of these autoantigens will shed light on the pathomechanisms involved in oMMP.

## Figures and Tables

**Table 1:** Information on patient samples.

Condition	Age	Sex	IIF	BP180 ELISA	BP230 ELISA	COL7 ELISA	Dsg1 ELISA	Dsg3 ELISA	ITGβ4 IB
MMP	51	F	+	+	−	−	−	−	*Nd*
	50	M	+	+	−	−	−	−	*Nd*
	86	F	−	+	−	−	−	−	*Nd*
	66	M	+	+	−	−	−	−	*Nd*
	37	F	+	+	−	−	−	−	*Nd*
	72	F	+	+	−	−	−	−	*Nd*
mcPV	54	M	+	−	*Nd*	*Nd*	+	+	*Nd*
	70	F	+	−	−	−	+	+	*Nd*
	66	M	+	*Nd*	*Nd*	*Nd*	+	+	*Nd*
BP	87	F	+	+	+	−	*Nd*	*Nd*	*Nd*
	*NR*	*NR*	+	+	+	−	−	−	*Nd*
	89	F	+	+	+	−	*Nd*	*Nd*	*Nd*
oMMP	*NR*	*NR*	*Nd*	−	−	*Nd*	*Nd*	*Nd*	+
	*NR*	*NR*	*Nd*	−	−	*Nd*	*Nd*	*Nd*	+
	*NR*	*NR*	*Nd*	−	−	*Nd*	*Nd*	*Nd*	+
	80	M	−	*Nd*	*Nd*	*Nd*	*Nd*	*Nd*	+
	73	F	+	*Nd*	*Nd*	*Nd*	*Nd*	*Nd*	+
	82	M	+	*Nd*	*Nd*	*Nd*	*Nd*	*Nd*	+
	63	F	+	−	−	*Nd*	*Nd*	*Nd*	−
	*NR*	*NR*	−	−	−	*Nd*	*Nd*	*Nd*	+
	*NR*	*NR*	−	−	−	*Nd*	*Nd*	*Nd*	+
	*NR*	*NR*	−	−	−	*Nd*	*Nd*	*Nd*	+
	78	M	−	−	−	*Nd*	*Nd*	*Nd*	+
	83	M	−	−	−	*Nd*	*Nd*	*Nd*	−

BP—bullous pemphigoid; mcPV—mucocutaneous pemphigus vulgaris; IB—immunoblotting; IIF—indirect immunofluorescence; MMP—mucous membrane pemphigoid; Nd—not done; NR—not recorded; oMMP—ocular predominant mucous membrane pemphigoid.

**Table 2:** Top 20 targeted autoantigens in oMMP using PhIP-seq.

Gene	Protein Name	*p*	oMMP (%)	Controls (%)
*TNKS1BP1*	182 kDa tankyrase-1-binding protein	0.003	83	16
*SEC16B*	Protein transport protein Sec16B	0.004	58	0
*DENND1C*	DENN domain-containing protein 1C	0.004	58	0
*FNBP4*	Formin-binding protein 4	0.004	58	0
*CASZ1*	Zinc finger protein castor homolog 1	0.009	66	8
*GOLGB1*	Golgin subfamily B member 1	0.009	66	8
*ARHGEF7*	Rho guanine nucleotide exchange factor 7	0.009	66	8
*DOT1L*	Histone-lysine N-methyltransferase, H3 lysine-79 specific	0.009	66	8
*FAT3*	Protocadherin Fat 3 isoform 2 precursor	0.012	75	16
*ARHGAP6*	Rho GTPase-activating protein 6	0.012	83	25
*SRRT*	Serrate RNA effector molecule homolog	0.013	50	0
*RPL6*	60S ribosomal protein L6	0.013	50	0
*LARP4B*	La-related protein 4B	0.013	50	0
*PRDM15*	PR domain zinc finger protein 15	0.013	50	0
*ELOA2*	Elongin-A2	0.013	50	0
*FCGRT*	IgG receptor FcRn large subunit p51	0.013	50	0
*CLASP2*	CLIP-associating protein 2	0.013	50	0
*HIP1*	Huntingtin-interacting protein 1	0.027	58	8
*CILP*	Cartilage intermediate layer protein 1 preproprotein	0.027	58	8
*NAV2*	Neuron navigator 2	0.036	83	33

## Data Availability

The original contributions presented in the study are included in the article/Supplementary Material, further inquiries can be directed to the corresponding author.

## Supplementary Materials

The following supporting information can be downloaded at https://www.mdpi.com/article/10.3390/antib13040091/s1, Table S1: oMMP autoantibodies versus other AIBDs; Table S2: BP autoantibodies versus other AIBDs; Table S3: MMP autoantibodies versus other AIBDs; Table S4: mcPV autoantibodies versus other AIBDs; Table S5: Heatmap matrix; Table S6: oMMP autoantibodies versus other AIBD proportions; Table S7: oMMP autoantibodies versus other AIBD GO analyses; Table S8: mcPV autoantibodies versus other AIBD proportions.
